# Impact of omega-3 fatty acids on calorie intake and certain anthropometric measurements in children with sickle cell disease in Saudi Arabia

**DOI:** 10.6026/97320630015189

**Published:** 2019-03-15

**Authors:** Shahida Khan, Ghazi Damanhouri, Tahir Jameel, Ashraf Ali, Ahmad Makki, Sarah Khan, Ibtehal AlAnsari, Saeed Halawani, Fatma Zahrani, Mohammad AlKazmi, Ibrahim Ghita

**Affiliations:** 1 Applied Nutrition Group, King Fahd Medical Research Center, King Abdulaziz University, Jeddah, Kingdom of Saudi Arabia; 2 Department of Medical Laboratory technology, Faculty of Applied Medical Sciences, King Abdulaziz University, Jeddah, Kingdom of Saudi Arabia; 3 Department of Hematology, King Abdulaziz University hospital, King Abdulaziz University, Jeddah, Kingdom of Saudi Arabia; 4 King Abdulaziz University hospital, Hematology department, Rabigh Branch, Kingdom of Saudi Arabia; 5 Diabetes Unit, King Edward Memorial Hospital, 489, Sardar Moodliar Road, Rasta Peth, Pune, India; 6 Department of Hematology and Immunology, Umm Al Qura University, Faculty of Medicine, Makkah, Kingdom of Saudi Arabia; 7 Department of Pediatrics, King Abdulaziz University hospital,King Abdulaziz University, Jeddah, Kingdom of Saudi Arabia; 8 Department of Hematology, Al-Noor Specialist hospital Makah, Kingdom of Saudi Arabia

**Keywords:** Diet, nutrition deficiency, disease severity, sickle cell disease, Saudi Arabia

## Abstract

The nutritional status and growth in children with sickle cell disease is compromised due to intake of diet that is low in calories as well as deficient in nutrients. Growth stunting and a low body mass index have been observed in these children. Some children exhibit pica, which is an abnormal eating pattern by ingesting things other than food, like paper, wood etc. This also was found to correlate to lower hemoglobin values. Interventions with certain essential nutrients such as omega-3 fatty acids are known to benefit these children in terms of lowering their complications due to the disease. We therefore wished to see if omega-3 fatty acids exhibit positive effects on their nutritional intake and growth parameters too. Hence, we supplemented these children with omega-3 fatty acids for a period of six months. Both the male and female children with the disease significantly improved their calorific intake as well as body mass index. Also a lowering of pica status was distinctly observed.

## Background

The Kingdom of Saudi Arabia is majorly a consumer based society, reliant on convenience foods due to its dependency on imports from around the globe. Consumption of nutrition deficient fast foods and wrong eating habits are the major principal causes of most of the diseases like blood pressure, diabetes, dental problems, anemia etc, that are prevalent and growing at a fast pace today. Junk food appears to be prevalent across all ages and these children are no exception [1]. It is widely accepted that sickle cell disease (SCD) children exhibit growth retardation during childhood and early youth [2]. Earlier researchers have observed underweight and stunting in children with sickle cell disease when compared with normal healthy children [3]. The determinants of low growth in these children could probably be due to various reasons like genetics factors phenotypic polymorphism, low fetal hemoglobin levels, environmental factors, and specific nutrient deficiencies [4]. Under nutrition leading to poor growth is markedly seen in almost all the children affected by sickle cell disease [2], 5]. Hypermetabolism associated with childhood SCD, accounts for the typical hemolytic anemia [6]. Consequently, nutrient deficiency being concomitant to calorific demand and energy output has resulted in immune-compromised children with all round growth deficiency [7]. Reduced nutrient absorption and increased utilization, has been found to be inversely associated with the level of anemia severity [8]. The increased requirements of energy in sickle cell disease, causes growth deficits reflected in lowered weights, heights and a lowered body mass index (BMI). Dysfunctional eating (or pica) appears to be underreported being prevalent in 56.4% to 66.2% of children and adolescents with SCD as compared to 40 % in controls [9]. Since the late 1980's, nutrition deficiency has been reviewed as a serious feature of SCD that demands it to be considered as an essential part of clinical care [10]. Hydroxy-urea shows improvements by increasing fetal hemoglobin levels, but its association with certain side effects make its administration cautious [11].

A small successful dietary intervention was carried out with five children with HbSS, aged 3-16 years way back in 1985 .Though these children affected with growth retardation showed clinical improvements, data interpretation was difficult as four different protocols of supplementation were used [12]. Nevertheless this paved the way for future supplemental researches. Several trials have also been conducted with zinc, glutamine etc; to enhance growth parameters and recompense for hyper metabolism linked to this disease [13].

The essential vitamin folic acid is widely administered to patients with SCD for the production of RBCs [14]. A vitamin D supplementation study conducted showed a positive outcome by researchers at Emory University [15]. Pilot trials with combined nutritional therapy of macro and micronutrients, with synergistic effects proved more effective in improving health outcomes for providing further calories in a low volume package that also appealed to children [16]. Many trials in SCD patients supplemented with the essential omega-3 fatty acids show a reduction in the severity of anemia, white blood cell count, vasoocclusive pain episodes and pro-thrombotic activity [17]. The supplementation would therefore be expected to improve nutritional as well as growth parameters. We therefore envisaged assessing the improvement in calorific intake correlating with growth parameters in children with SCD from Saudi Arabia by dietary supplementation with omega-3 fatty acids. 

## Methodology

### Study Population:

The study was performed at the Applied Medical Nutrition Center of King Fahd Medical Research Center, King Abdul-Aziz University in Jeddah. Of the eighty six children enrolled, sixty six children filled the nutritional dockets, with 35 females and 31 males between the ages 5-16 years old. Of these 24 children (14 males and 10 females) undertook the omega-3 supplementation for a period of 6 months. Consent forms were obtained from guardians for enrolment into the program. Medical reports of all patients were duly collected from the respective clinicians. Patients who had not undergone painful crisis for the past one month and in a steady state were included in the study. 

### Anthropometric measurements:

Height measurements of all children enrolled was done using a stadio-meter. Weight, BMI, and body fat percent were measured using a TANITABC 418 equipment (BC-418MA, Tanita Corporation, Tokyo, Japan). Skin fold thickness and waist hip ratio could not be taken due to non-compliance of the conservative patient families.

### Dietary Intake Assessment:

A daily diet record comprising of a comprehensive food and beverage consumption of all patients during a day, (including breakfast, lunch, dinner, in-between snacks, and water intake) was recorded after consultation with their guardians. The calories of all the local dishes were calculated according to earlier works done [18]. Calculation of nutrient intake was performed before and after six months of omega 3 fatty acid supplementation. Average calories of age and gender were calculated based on the values published by the Institute Of Medicine dietary reference intakes USA [19]. Based on their weights, the patients were assigned one or two teaspoon dose of omega-3 syrup (Brain Wise from KADSUN Healthcare Pvt. Ltd, Pune, India). Children less than 25 kg body weight were given one teaspoon per day, and those 25 kg and above were supplemented with 2 teaspoons per day) Each teaspoon would contain 190 mg of DHA the potent omega-3 fatty acid. Hemoglobin was estimated using an automated hematology analyzer.

### Data Analysis

Statistical analysis expressed the results as means ± along with the standard deviations. A t-test was performed and comparison of means done statistically, with the significant values noted for p<0.05

## Results

### Calorific intake:

A six month oral supplementation course of omega-3 fatty acids showed improvement in the quantity of nutritional intake in males and females of both the age groups, as observed in table. Males show only a slightly more improvement than the females with a p value of 0.00024 as compared to 0.000970 for females which is minimal. Both age groups too showed significant increases in consumption of calories after omega-3 supplementation, with p values of 0.017 for the age group 5-10 yr and 0.0011 for the 11-16 yr age group. When compared with the required amount of calories for their ages, deficits in calorie intake reduced on supplementation with omega-3 fatty acids as seen in Table 1.

A significant reduction in the dysfunctional eating was also noticed on supplementation with omega 3 fatty acids. 13% of the children were affected by this behavior of which tissue or paper eating comprised 6%, nail biting and eating 5%, and sand eating 2%. Dysfunctional eating disappeared completely after the six month supplementation. All cases of eating paper nails and sand were reported evanescent of that behavior after omega-3 supplementation, with a p value of 0.000, as seen in Table 2.

### Growth parameters:

Physical characteristics of all children undergoing the six month supplementation with omega-3 fatty acids, significantly improved with increased height (p value 0.009), and weight increase (p value 0.0043). BMI of these patients also showed improvement though not significant with a p value of 0.37. All patients supplemented with omega-3 fatty acids showed improvement in body fat percent with a p value of 0.01which is seen in Table 3. The concentration of hemoglobin also showed a significant increase with p value of 0.045.

## Discussion and Conclusion:

Nutritional shortcomings and deficiencies are one of the major factors responsible for the compromised growth, wasting and stunting of children with sickle cell disease. Low food consumption, anorexia, health complications are all contributory and collateral to the observed poor growth. Intake of food lowers calorific as well as nutrient value, poor absorption and decreased utilization by the body were noted by earlier researchers [5]. A point noted in our study was that all children were eating fast food /junk food. Therefore, no healthy calories were ingested. The outcome shows that nutrient dense foods should be consumed in order to meet the demands of their bodily metabolic processes. The disease condition is beset with complications like repeated infections and vaso-occlusive crisis pain causing a disinterest in food. This may give rise to both mother and child taking recourse to convenience foods. The need to educate the mother on refraining from ready to eat foods for the family, and especially children with sickle cell disease is of great importance. Anorexia is a feature observed in many children with the disease, which could result from the pain, frequent hospitalizations and thereby decreased energy for various activities. This leads to a significant lowering in food intake. In particular, children with SCD are frequently choosy and do not easily adapt to a new diet. Our study revealed that, in the Saudi population, inadequate nutritional intake, is ironically on account of ample fast food choice as seen in. Researchers from Saudi Arabia have reported stunted and compromised growth patterns in SCD patients which appear to be associated with the severity of the disease [20], which is also observed in our study (Table 3) warranting a second look into nutritional therapies.

A lowering in the number of pain episodes associated with the disease reflected in better appetite and higher intake of calories as observed by us. Omega-3 fatty acids are precursors of bioactive compounds and exhibit multifaceted roles in, cell signaling, enhancing membrane fluidity, receptor functioning, and preventing oxidative damage. Therefore their affectivity on a multifaceted disease like sickle cell disease would seem very apt. These were supplemented by us in a fruit flavored syrup form, which was obviously very palatable as they showed an immediate liking for it. This helped the effortless adaptation to the product. This led to the children in our study feeling hungrier and eager not only to eat food but a healthy one at that which helped ease of administration.

A positive attitude towards their well-being, and dietary consumption resulted from the omega-3 supplement. A low hemoglobin count renders more energy demands on the body thus decreasing the growth parameters. Supplementation with the healthy fat omega-3 resulted in improved Hb percentage and highly improved growth parameters of BMI and body fat percent, which can be noticed in Table 3.

In our study, following the supplementation with omega-3 fatty acid syrup, pica behavior disappeared in all the affected children, linking to improvement in nutrition and wellness as observed in Table 2. Earlier studies have linked association of pica behavior with social emotional stress, anxiety, and depression [21]. Omega-3 fatty acids are known to be effective in cases of depression, anxiety and other related mental health disorders [22]. The improvement in mood reflects in the improvement in pica behavior. This beneficial effect of omega-3 fatty acids as seen in our study has not been reported earlier. Supplementation with the healthy fat omega-3 fatty acids results in better appetite, and physical health, which calls for omega-3 fatty acids to be used as a therapeutic measure in these children. Dietary supplementations should therefore be considered as an integral means to the treatment regimen of this disease. Limited studies on calorie intake and growth parameters have been done in Saudi Arabia, and this would give impetus for future structured researches on nutritional status in children with SCD.

## Conflict of Interest

Authors declare no conflict of interest

## Figures and Tables

**Table 1:** Calories consumed according to ages and gender on supplementation with omega-3 fatty acids

Avg.Calories Consumed Kcal/day	No. of totalpatients 'n'	Pre Supplementation	Post Supplementation	Recommended calories	p- value
Male + female	24	1285.076±SD369.06	1534.872±SD382.22	1850	3.87E-07
Male	14	1357.12±SD 399.19	1611.455±SD 409.81	1900	0.00024
Female	10	1184.215±SD 297.98	1427.656±SD 313.03	2200	0.00097
5-10 years	16	1354.71±SD 338.16	1616.66±SD 334.503	1850	<0.001
11-16 years	8	1115.93±SD 359.88	1336.25±SD 390.65	2200	<0.009

**Table 2:** Dysfunctional eating in children with sickle cell disease

n	percent	Pre- Supplementation	Post-Supplementation	percent	p value
Total	66	100				
Dysfunctional eating	13	19.696	13	0	0	0
Paper/tissue	6	9.09	6	0	0	0
nails	5	7.575	5	0	0	0
sand	2	3.03	2	0	0	0

**Table 3:** Growth parameters in children with sickle cell disease supplemented with omega-3 fatty acids

Gender Age groups	Height	Weight	BMI	BF (%)	Hb	
			Pre Supplementation	Post Supplementation	Pre Supplementation	Post Supplementation	Pre Supplementation	Post Supplementation	Pre Supplementation	Post Supplementation	Pre Supplementation	Post Supplementation
Male		mean	120.43	125.93	23.01	24.77	15.26	14.97	18.04	14.97	7.25	7.85
	14	SD	16.21	17.19	9.79	10.73	2.71	2.71	9.36	2.71	0.98	1.41
		P value		0.004		0.012		0.537		0.193		0.134
Female		mean	131.1	134	26.78	24.9	15.16	14.99	18.54	14.99	7.35	7.86
	10	SD	22.99	23.31	9.23	9.69	1.92	1.84	6.39	1.84	0.81	2.34
		P value		0.002		0.013		0.295		0.088		0.445
5 to 10yrs		mean	114.56	117.69	19.89	20.17	14.84	14.78	18.78	14.78	7.31	7.63
	16	SD	11.03	11.57	5.7	6.09	2.22	2.42	8.38	2.42	0.95	1.73
		P value		0.000		0.044		0.798		0.052		0.398
11 to 16yrs		mean	145.5	152.5	33.98	35.65	15.99	15.39	17.21	15.39	7.26	8.31
	8	SD	16.59.58	8.85	9.57	2.6	2.27	8.2	2.27	0.84	1.99
		P value	0.037		0.008		0.407		0.505	0.198

## References

[1] https://www.moh.gov.sa/en/HealthAwareness/EducationalContent/Food-and-Nutrition/Pages/Poor_Nutrition.aspx.

[2]  Hyacinth HI (2013). J Soc Behav Health Sci..

[3]  Lukusa KA (2017). Anemia..

[4]  Aloni, MN, Nkee L (2014). Hemoglobi..

[5]  Al-Saqladi AW (2010). Ann Trop Paediatr..

[6]  Mandese V (2015). Nutrition Journal.

[7]  Singhal A (2002). Am J Clin Nutr..

[8]  Kawchak DA (2007). J Am Diet Assoc..

[9]  Fathelrahman EA (2015). Basic Research Journal of Medicine and Clinical Sciences..

[10] Tomer A (2001). Thromb Haemost..

[11] Steinberg MH (2010). Am J Hematol..

[12] Heyman MB (1985). The Lancet..

[13] Williams R (2004). J Pediatr Hematol Oncol..

[14] Dixit R (2016). Cochrane Database Syst Rev..

[15] Osunkwo I (2011). Br J Haematol..

[16] Chan AC (2000). Nutrition.

[17] Daak AA (2013). Am J Clin Nutr..

[18] https://www.moh.gov.sa/en/HealthAwareness/Campaigns/badana/Pages/009.aspx.

[19] https://health.gov/dietaryguidelines/2015/guidelines/appendix-2/.

[20] El-Hazmi MA (2011). Indian J Med Res..

[21] O'Callaghan ET, Gold JI (2012). Journal of Pediatric Nursing: NursingCare of Children andFamilies.

[22] Larrieu T, Lav�S (2018). Front Physiol..

